# TWIST2 and the PPAR signaling pathway are important in the progression of nonalcoholic steatohepatitis

**DOI:** 10.1186/s12944-021-01458-0

**Published:** 2021-04-20

**Authors:** Yanmei Zhang, Xiaoxiao Ge, Yongqing Li, Bingyang Zhang, Peijun Wang, Mingju Hao, Peng Gao, Yueyi Zhao, Tao Sun, Sumei Lu, Wanshan Ma

**Affiliations:** 1 Department of Laboratory Medicine, Shandong Provincial Qianfoshan Hospital, Shandong University, Jinan, Shandong 250014 P. R. China; 2 Department of Clinical Laboratory, the Affiliated Hospital of Shandong University of Traditional Chinese Medicine, Jinan, Shandong 250014 P.R. China; 3 Department of Laboratory Medicine, The First Affiliated Hospital of Shandong First Medical University, Jinan, Shandong 250014 P. R. China; 4 Medical Research Center, The First Affiliated Hospital of Shandong First Medical University, Jinan, Shandong 250014 P. R. China

**Keywords:** Nonalcoholic fatty liver disease, Hepatocyte steatosis, TWIST family bHLH transcription factor 1 (TWIST1), TWIST family bHLH transcription factor 2 (TWIST2), Peroxisome proliferator activated receptor gamma (PPARγ), The PPAR signaling pathway, High-fat diet

## Abstract

**Background:**

To investigate the roles of the transcription factors twist family bHLH transcription factor 1 (TWIST1), twist family bHLH transcription factor 2 (TWIST2), and peroxisome proliferator activated receptor gamma (PPARγ) in the progression of nonalcoholic steatohepatitis.

**Methods:**

The protein levels of TWIST1, TWIST2 and PPARγ were determined in the serum of nonalcoholic fatty liver disease (NAFLD) patients and healthy controls by enzyme-linked immunosorbent assay (ELISA). An in vivo model for fatty liver was established by feeding C57BL/6 J mice a high-fat diet (HFD). An in vitro model of steatosis was established by treating LO-2 cells with oleic acid (OA). RNA sequencing was performed on untreated and OA-treated LO-2 cells followed by TWIST1, TWIST2 and PPARγ gene mRNA levels analysis, Gene Ontology (GO) enrichment and pathway analysis.

**Results:**

The TWIST2 serum protein levels decreased significantly in all fatty liver groups (*P* < 0.05), while TWIST1 varied. TWIST2 tended to be lower in mice fed an HFD and was significantly lower at 3 months. Similarly, in the in vitro model, the TWIST2 protein level was downregulated significantly at 48 and 72 h after OA treatment. RNA sequencing of LO-2 cells showed an approximately 2.3-fold decrease in TWIST2, with no obvious change in TWIST1 and PPARγ. The PPAR signaling pathway was enriched, with 4 genes upregulated in OA-treated cells (*P* = 0.0018). The interleukin (IL)-17 and tumor necrosis factor (TNF) signaling pathways were enriched in OA-treated cells.

**Conclusions:**

The results provide evidence that the TWIST2 and PPAR signaling pathways are important in NAFLD and shed light on a potential mechanism of steatosis.

## Introduction

Nonalcoholic fatty liver disease (NAFLD) is a common chronic liver disorder characterized by lipid accumulation in hepatocytes. Currently, NAFLD affects approximately 20–30% of the Western population and 5–18% of the Asian population and is continuing to increase [1]. Moreover, being a metabolic disease, NAFLD can aggravate other metabolic syndromes, including obesity, type 2 diabetes mellitus, dyslipidemia, hypertension and insulin resistance (IR) [2, 3]. However, the pathogenesis and molecular mechanism of NAFLD are still unclear, and further work is undoubtedly necessary.

NAFLD includes simple steatosis as well as nonalcoholic steatohepatitis (NASH), a more serious condition with associated inflammation. It is well known that NASH can progress to more serious conditions, such as fibrosis, cirrhosis and hepatocellular carcinoma [4]. The widely accepted pathogenesis of NAFLD is the “multiple hit theory”, which suggests that in the development of NAFLD, many hits might work together in parallel [5–7]. Obesity and IR are reported to play a fundamental role in the progression of NAFLD. High insulin levels promote lipid synthesis by increasing glycolysis in the liver [8]. In addition, high insulin also reduces the expression of ApoB-100 and microsomal triglyceride transfer protein (MTTP), leading to a reduction in very low density lipoprotein (VLDL) output in the liver, eventually leading to lipid deposition in the liver tissue [9]. IR can also cause hepatic steatosis, and it has been proposed that hepatic steatosis further aggravates hepatic insulin resistance, creating a feedback loop between the two conditions [10]. Kim et al. suggested that hepatic steatosis could lead to IR, but some scholars believe that hepatic steatosis is not enough to cause liver IR [11, 12]. Recently, much more is said about molecular mechanisms because the mechanisms that lead to the release of many mediators are known, which then lead to the formation of proliferation and scar tissue after a long inflammatory process, induction of apoptosis and/or necrosis [13]. The relationship between hepatic steatosis and IR is still controversial and needs further study.

The authors’ research team has previously reported that two transcription factors, TWIST1 and PPARγ, have a positive regulatory role in the insulin sensitivity of 3 T3-L1 adipocytes [14]. In IR models of 3 T3-L1 adipocytes and C57/BL6J mice, silencing TWIST1 expression can relieve IR to a certain degree, indicating the potential clinical value of TWIST1 and PPARγ in steatosis-related disease [15]. As hepatocytes are target cells of insulin, the role of TWIST1 and PPARγ in hepatocytes is undoubtedly worth further exploration. At the same time, TWIST2, the other member of the TWIST family, attracted attention for its role in metabolism and metabolic diseases. Overexpression of TWIST2 was reported to ameliorate hepatocellular steatosis and inhibit inflammation [16].

The present study will continue previous work to explore the role and possible mechanism of TWIST1, TWIST2, and PPARγ in the development of hepatocyte steatosis. This work will mainly be based on clinical samples and in vivo and in vitro models of hepatocyte steatosis. The results will provide evidence for the role of TWIST and PPARγ in NAFLD, clarify their function in the adipogenesis process and provide new insights into steatosis-related diseases.

## Materials and methods

### Patient inclusion and human serum sample collection

A total of 406 individuals who had a health check-up at Shandong Provincial Qianfoshan Hospital from December 2017 to December 2018 were recruited into the present study. Following the inclusion criteria, these NAFLD patients were generally 18–60 years old, had normal thyroid function, no history of drinking, no malignancy, no viral hepatitis, no drug-induced liver disease, no autoimmune liver disease, and all other specific diseases that cause fatty liver.

NAFLD patients were divided into mild, moderate, and severe NAFLD groups based on abdominal ultrasound testing. The standard of ultrasound fatty liver classification is mainly based on the shape of the liver, the echo of the liver parenchyma, the degree of attenuation behind the liver, and the visualization of the intrahepatic duct. Generally, patients with mild fatty liver have a normal liver shape and a sharp angle. In patients with moderate fatty liver, the liver is slightly larger, and the angle becomes blunt. In patients with severe fatty liver, the liver is obviously enlarged, and the angle is round and blunt. The liver parenchyma echoes, the patients with mild fatty liver are fine and thicker, and the echoes of patients with severe fatty liver are obviously thicker. The attenuation at the rear of the liver changes from normal to obvious, and the intrahepatic ducts change from normal to unclear. Different grades can distinguish mild, moderate and severe fatty liver. The condition was diagnosed by the ultrasound physician. Healthy adults who were also having a health check-up at the same time had samples randomly collected. This study was performed in accordance with ethical standards and was approved by the Ethics Committee of Shandong University (No: [2017] S048).

### Materials and reagents

C57/BL6 mice were purchased from the Experimental Animal Center of Shandong University. The high-fat diet (No: D12492) and basal diet (No: D12450B) were both products of Research Diets (New Brunswick, USA). The human hepatic cell line LO-2 (also named HL-7702) was purchased from Procell Life Science & Technology Co., Ltd. (Wuhan; China). The human TWIST1, TWIST2 and PPARγ ELISA kits were Jianglai Biology (Shanghai, China) products. RPMI-1640 medium, fetal bovine serum (FBS) and 0.25% trypsin-0.02% EDTA were all purchased from GIBCO (Invitrogen, California, USA). The rabbit anti-mouse TWIST1 mAb was purchased from Sigma (St. Louis, MO, USA). Rabbit anti-rabbit TWIST2 pAb and rabbit anti-mouse PPARγ mAb were purchased from Abcam (Cambridge, MA, USA). The rabbit anti-mouse β-actin mAb and secondary antibodies, including horseradish peroxidase-conjugated anti-mouse IgG for TWIST1, PPARγ, and β-actin and horseradish peroxidase-conjugated anti-rabbit IgG for anti-TWIST2, were purchased from ZSGB-BIO (Beijing, China). Oleic acid (OA) and all other general reagents used in this study were purchased from Sigma (St. Louis, MO, USA).

### Serum determination of TWIST1, TWIST2 and PPARγ in patients

The levels of TWIST1, TWIST2 and PPARγ in the serum of patients were analyzed by ELISA per the manufacturer’s instructions. Generally, standard or serum (each 50 μL) was added to the plate wells; HRP-conjugated antibody (100 μL) was added to the plate to mix with standard or serum at 37 °C for 1 h. After being washed 5–6 times, the plates were treated with 50 μL each of substrates A and B and incubated at 37 °C for 15 min, and then 50 μL of terminating buffer was added to stop the reaction. Finally, the OD value was checked under an enzyme-linked immunoassay analyzer at 450 nm.

### Induction of NAFLD in C57/BL6 mice

Sixty-four male C57/BL6 mice (6 weeks of age) were included in the study and housed 6 per cage. The mice were housed in a specific environment: 25 °C, 55% relative humidity, cycle of 12 h light and 12 h dark. They were free to eat and drink tap water ad libitum. After a week, they were randomly assigned to two groups, the control group (basal diet, 4% fat, with 20 g% lard) and the high-fat diet (HFD) group (HFD, 60% fat, with 225 g% lard). They were fed a control diet or HFD for 4, 8, 12, or 16 weeks, and their body weights were recorded every week. After the mice were euthanized, liver tissues were collected and snap frozen. Hematoxylin and eosin (H&E) staining and Oil red O staining were conducted on liver sections. The additional procedures were carried out according to the ‘Principles of Laboratory Animal Care’ established by the National Institutes of Health. This study was performed in accordance with ethical standards and was approved by the Ethics Committee of Shandong University (No: [2017] S048).

### Intraperitoneal glucose tolerance test (IPGTT) and intraperitoneal insulin tolerance test (IPITT)

Before the mice were sacrificed at the 16th week, IPGTT and IPITT were conducted in a portion of the mice to determine the ability of mice to respond to glucose and insulin. After fasting overnight for IPGTT or six hours for IPITT, 12 mice were injected with 50% glucose (2.0 g/kg, i.p.), and 12 mice were injected with insulin (0.65 U/kg, i.p). Blood glucose levels in the mice were analyzed by means of a glucose test strip (Bayer, Germany) after blood samples were taken via the caudal vein at 0, 30, 60, 90, 120, 150 and 180 min after glucose or insulin injection. The area under the curve (AUC) was compared among different groups to analyze the IPGTT and IPITT.

### Establishment of the LO-2 hepatocyte steatosis model

LO-2 hepatocytes were cultured in RPMI 1640 supplemented with 10% fetal bovine serum, 100 U/mL penicillin and 100 μg/mL streptomycin. The cells were cultured in a humidified chamber at 37 °C and 5% CO_2_. For steatosis induction, RPMI-1640 supplemented with 10% FBS and OA (50 μg/mL) was added to the cells when they reached approximately 60% confluence. The culture medium and OA were replaced every 24 h for 24, 48, 72, and 96 h.

### Hematoxylin-eosin (H&E), oil red O staining of frozen liver sections and LO-2 hepatocytes

After the mice were sacrificed and the livers were removed and snap frozen, H&E staining was performed on frozen sections. The general steps are as follows: Methanol fixative for 60 s; Hematoxylin 2 min; Washed with phosphate buffered saline (PBS) for 30 s; Hydrochloric acid alcohol washed for 3 s; Washed with PBS for 30 s; Lithium carbonate 10 s; Washed with PBS for 30 s; 90% alcohol for 10 s; Eosin for 5 s; 90% alcohol for 5 s; 95% alcohol for 20 s, twice; 100% alcohol for 20 s; 100% alcohol for 20 s; Xylene for 30 s, twice. The resulting slides were visualized with an inverted phase contrast microscope.

For Oil red O staining, LO-2 cells were grown on cover slips, and after removal of cell culture medium, cells were washed three times with PBS and fixed with 4% tissue cell fixative solution (Solarbio Science) at room temperature for 10 min. Frozen slices for hepatic tissue were similarly fixed. After washing three times with PBS, the cells or frozen slices were stained with freshly diluted Oil red O solution (0.1% Oil red O dissolved in 60% isopropyl alcohol and 40% distilled water) for 15–30 min. Cells or frozen slices were then washed with 60% isopropyl alcohol for 1 min and washed with PBS three times. Images were obtained using an inverted phase contrast microscope. Oil red O staining was quantified by an absorbance assay. Briefly, the Oil red O stain was solubilized with isopropyl alcohol and the optical density at 510 nm was measured by spectrophotometry (Multiskan Go, Thermo Scientific). All experiments were conducted in triplicate.

### Protein extraction and western blotting analysis

Total protein was extracted using radioimmunoprecipitation assay (RIPA) lysis buffer containing protease inhibitor cocktail (Thermo Scientific, USA) and phosphatase inhibitor cocktail (Thermo Scientific, USA), and the protein concentration was analyzed by Pierce™ BCA protein assay as previously shown [14, 15]. The protein (40 μg for each sample) was resolved on SDS-PAGE gels and transferred to a Hybond-P PVDF membrane. The membranes were blocked with 5% milk or 5% bovine serum albumin (BSA) for 1 h. The membranes were incubated with primary antibodies (anti-TWIST1, 1:1000; anti-TWIST2, 1:1000; anti-PPARγ, 1:1000 and anti-β-actin, 1:2500) at 4 °C overnight. After washing three times (20 min each) with TBST, the secondary antibodies were incubated with horseradish peroxidase-conjugated anti-rabbit or anti-mouse (1:5000) antibodies for 1 h at room temperature. Blots were washed three times (20 min each), and image development was performed with electrochemiluminescence (ECL) reagent. All experiments were performed in triplicate.

### RNA sequencing analysis

RNA sequencing analysis was performed using the Illumina HiSeq 4000 platform (KangChen Bio-tech, Shanghai, China). Briefly, cultured LO-2 cells were treated with DMSO (the control group) and OA (50 μg/mL, 48 h). After treatment, cells were lysed with TRIzol reagent. RNA was extracted as normal protocol. RNA (1–2 μg) was used to establish the RNA library based on specific kits. It includes RNA fragmentation, primed 1st strand cDNA synthesis, dUTP-based 2nd strand cDNA synthesis, adaptor ligation and PCR amplification. The library was qualified using an Agilent 2100, quantified by qPCR, and then subjected to sequencing based on Illumina X-ten/NovaSeq. Three biological replicates were prepared in each group. Genes were considered differentially expressed if the expression difference between two groups showed a twofold change or greater (log_2_FC > 1.0, fold change). Gene Ontology (GO) enrichment analysis was performed on the http://geneontology.org website, and the enrichment scores for molecular function (MF), biological process (BP) and cellular component (CC) were analyzed. Pathway analysis for differentially expressed genes was further conducted based on the Kyoto Encyclopedia of Genes and Genomes (KEGG) database, whose website is https://www.genome.jp/kegg/.

### Statistical analysis

The data were analyzed using SPSS 24.0 software (mean ± SD). The correlations between TWIST1, TWIST2, PPARγ, body mass index (BMI), fasting glucose, fasting insulin, or homeostasis model assessment (HOMA) and fatty liver severity were analyzed by ANOVA. For western blotting, we used Image J to determine the relative gray values of each band by comparison with β-actin. A *t* test was used to compare the differences between two groups, and ANOVA was used to compare the differences between more than two groups. *P* < 0.05 was considered an indicator of significant difference.

## Results

### Analysis of TWIST1, TWIST2, and PPARγ protein levels in human serum samples

The serum samples were collected at the clinical laboratory. A total of 406 human serum samples were collected in this study. The samples were divided into four groups based on abdominal ultrasound testing, with a healthy control group (*n* = 99), mild NAFLD group (*n* = 102), moderate NAFLD group (*n* = 107), and severe NAFLD group (*n* = 98). The Materials and Methods section describes the criteria for determining whether a fatty liver is mild, moderate, or severe. Sex, age, height, and body weight were recorded for each individual, and the basic clinical characteristics of these individuals are shown in Table 1. They were further divided into four groups based on BMI (kg/m^2^), with thin group (BMI<18.5 kg/m^2^, *n* = 4), normal group (BMI = 18.5–23.9 kg/m^2^, *n* = 84), overweight group (BMI = 24–27.9 kg/m^2^, *n* = 137), and obesity group (BMI ≥ 28 kg/m^2^, *n* = 181).

**Table 1:** Basic information on the human serum samples (*n* = 406). A total of 406 human serum samples were collected. Each individual’s sex, age, height, and body weight were recorded and are shown in Table 1

Parameter	Number
Sex	
male	344
female	62
Age (years)	
18–44	301
45–59	105
BMI (kg/m^2^)	
< 18.5	4
18.5–23.9	84
24–27.9	137
≥ 28	181
Fatty liver	
healthy control	99
mild fatty liver	102
moderate fatty liver	107
severe fatty liver	98

*BMI* body mass index

As shown in Table 2, fasting blood glucose and insulin levels were determined by an automatic analyzer. Both blood glucose and blood insulin increased significantly with the severity of fatty liver disease (*P* < 0.05). Spearman rank correlation analysis showed that a positive correlation was found between the severity of fatty liver disease and BMI (*r* = 0.761, *P* < 0.001) and HOMA value (*r* = 0.607, *P* < 0.001). Then, TWIST1, TWIST2 and PPARγ levels were measured in the serum of patients using ELISA kits. For TWIST1, the levels in the mild and severe fatty liver groups decreased significantly (^△^*P < 0.05*, compared with healthy control) and increased significantly in the moderate fatty liver group (^△^*P < 0.05*, compared with healthy control). The levels of TWIST2 in all of the fatty liver groups decreased significantly (^△^*P < 0.05*, compared with healthy control). For PPARγ, compared with the control group, there were no significant changes in the mild fatty liver, moderate fatty liver, and severe fatty liver groups (*P* > 0.05).

**Table 2:** Data analysis for the human serum samples. The levels of BMI, glucose, insulin, HOMA value, Twist1, Twist2, and PPARγ were recorded. Statistical analysis was performed using ANOVA. *P* < 0.05 was considered to indicate a significant difference

Parameter	BMI (X ± SD)	glucose (mmol/L,^−^X ± SD)	insulin (mIU/L, ^−^X ± SD)	HOMA (^−^X ± SD)	Twist1 concentration (pg/mL, ^−^X ± SD)	Twist2 concentration (pg/mL, ^−^X ± SD)	PPARγ concentration (mmol/L, ^−^X ± SD)
Group							
healthy control	22.42 ± 2.54^□, ☆, ※^	4.98 ± 0.50^□, ☆, ※^	6.93 ± 3.12^□, ☆, ※^	1.54 ± 0.74^□, ☆, ※^	287.54 ± 264.02^□, ☆^	2.63 ± 2.26	258.88 ± 241.26
mild fatty liver	26.32 ± 2.45^△, ☆, ※^	5.35 ± 0.50^△, ☆, ※^	9.19 ± 4.18^△, ☆, ※^	2.19 ± 1.02^△, ☆, ※^	176.62 ± 166.08^△, ☆, ※^	2.00 ± 1.34^△^	227.84 ± 208.65
moderate fatty liver	28.25 ± 2.75^△, □, ※^	5.92 ± 1.59^△, □, ※^	11.82 ± 6.77^△, □, ※^	3.15 ± 2.07^△, □, ※^	380.39 ± 331.48^△, □, ※^	2.04 ± 1.37^△^	216.30 ± 177.04
severe fatty liver	31.30 ± 3.31^△, □, ☆^	6.29 ± 1.93^△, □, ☆^	15.29 ± 7.66^△, □, ☆^	4.20 ± 2.23^△, □, ☆^	279.26 ± 238.77^□, ☆^	2.06 ± 1.16^△^	225.72 ± 182.68

*BMI*: body mass index, kg/m^2^, *HOMA* homeostasis model assessment; ^△^*P* < 0.05, compared with healthy controls; ^□^*P* < 0.05, compared with mild fatty liver; ^✩^*P* < 0.05, compared with moderate fatty liver; ^※^*P* < 0.05, compared with severe fatty liver

### Characterization of the in vivo model of NAFLD

**Fig. 1:**
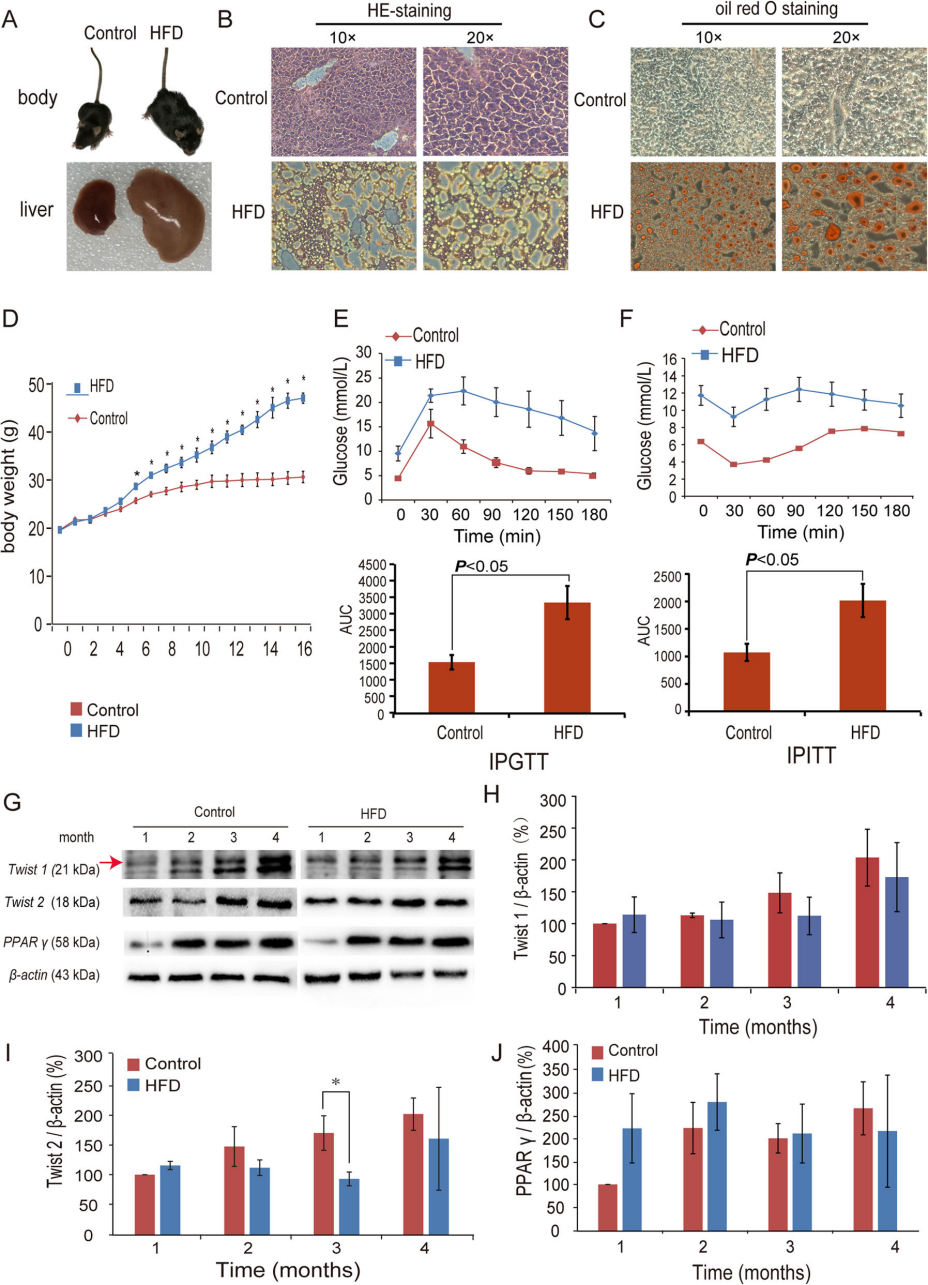
Characterization of an in vivo model of fatty liver disease and changes in TWIST1, TWIST2 and PPARγ protein expression in the liver (*n* = 8). **a** Comparison of the sizes of control and high-fat diet (HFD) group mice and their livers. **b** H&E staining of livers from control and HFD mice.**c** Oil red O staining of livers from control and HFD group mice. **d** Changes in body weight over time. **e** Intraperitoneal glucose tolerance test (IPGTT). **f** Intraperitoneal insulin tolerance test (IPITT). **g** Relative protein levels of TWIST1, TWIST2 and PPARγ in the livers of control and HFD group mice. The arrow shows that the upper band is the true Twist1. H/I/J) Semiquantitative assays of TWIST1 (**h**), TWIST2 (**i**), and PPARγ (**j**) in the livers of control and HFD group mice

After 16 weeks on a high-fat diet, the livers in the HFD group were enlarged and had a white hue (Fig. 1a). H&E staining (Fig. 1b) and Oil red O staining (Fig. 1c) showed increased lipid and triglyceride contents in the HFD group indicative of steatosis. The mice in the HFD group had a much higher body weight that increased significantly from 6 weeks of age (*P < 0.05, Fig. 1d). Both the IPGTT (Fig. 1e) and IPITT (Fig. 1f) of the HFD group showed a decreased insulin response. TWIST1, TWIST2 and PPARγ protein levels in liver tissues all changed with feeding time (Fig. 1g). Both TWIST1 (the arrow showed the true band) and TWIST2 expressions in HFD tended to be decreased in the detection, and statistical significance showed only in TWIST2 at 3 months after the start of the HFD (Fig. 1h, i). There were no significant change in PPARγ expression (Fig. 1j).

### Characterization of the in vitro model of hepatocyte steatosis

**Fig. 2:**
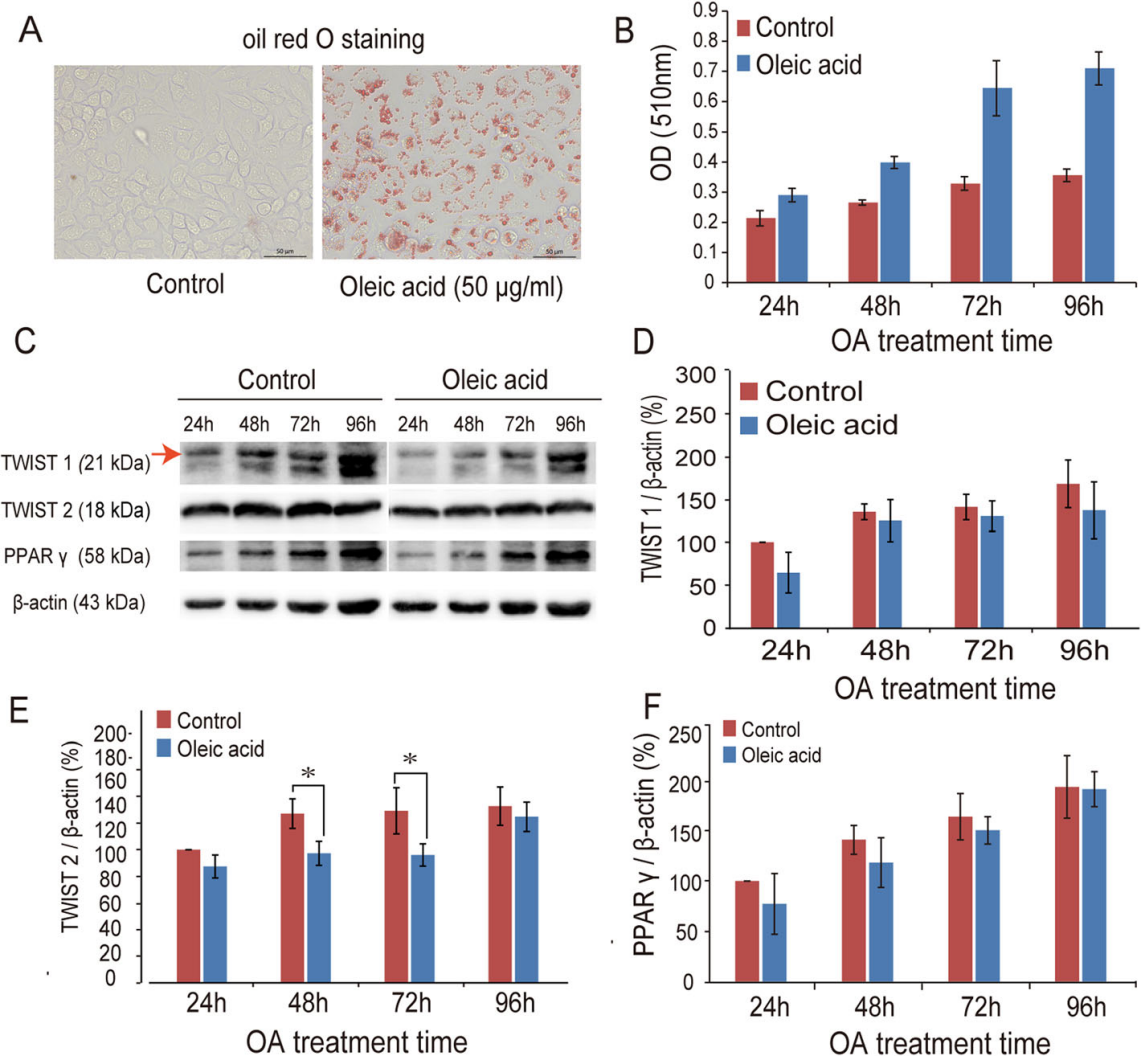
The LO-2 steatosis model shows increased lipid uptake and decreased TWIST2 expression. **a** Oil red O staining in the control and oleic acid (50 μg/mL) groups. **b** OD values for oil red O staining in the control and oleic acid groups. **c** TWIST1, TWIST2 and PPARγ expression in the control and oleic acid treatment groups in cultured LO-2 cells. β-Actin was used as a loading control. The arrow shows that the upper band is the true Twist1. **d**/**e**/**f**: Semiquantitative assays of TWIST1 (**d**), TWIST2 (**e**), and PPARγ (**f**)

LO-2 cells were treated with OA (50 μg/mL) for 24, 48, 72, and 96 h to induce steatosis. Obvious red-stained lipid droplets could be seen with Oil red O staining compared with the control (Fig. 2a, b), showing that the LO-2 cell steatosis model was established.

As shown in Fig. 2c, both TWIST1 (the arrow showed the true band) and PPARγ protein expression gradually increased with time (24, 48, 72, and 96 h) under OA treatment in LO-2 cells. However, similar increases could be found in control cells without OA exposure. The increased cell density may be the possible cause of TWSIT1 and PPARγ changes in LO-2 control cells under no external stimulation. Statistical analysis based on semi-quantitative analysis using Image J software showed that Both TWIST1 (Fig. 2d, the arrow showed the true band) and TWIST2 (Fig. 2e) in OA treatment tended to be decreased, and a significant difference could be found in TWIST2 at both 48 and 72 h. No significant change in PPARγ (Fig. 2f) expression between the control and OA (50 μg/mL) treatment groups (*P* > 0.05).

### RNA sequencing analysis and RT-PCR both showed that TWIST2 was decreased significantly by OA treatment

**Fig. 3:**
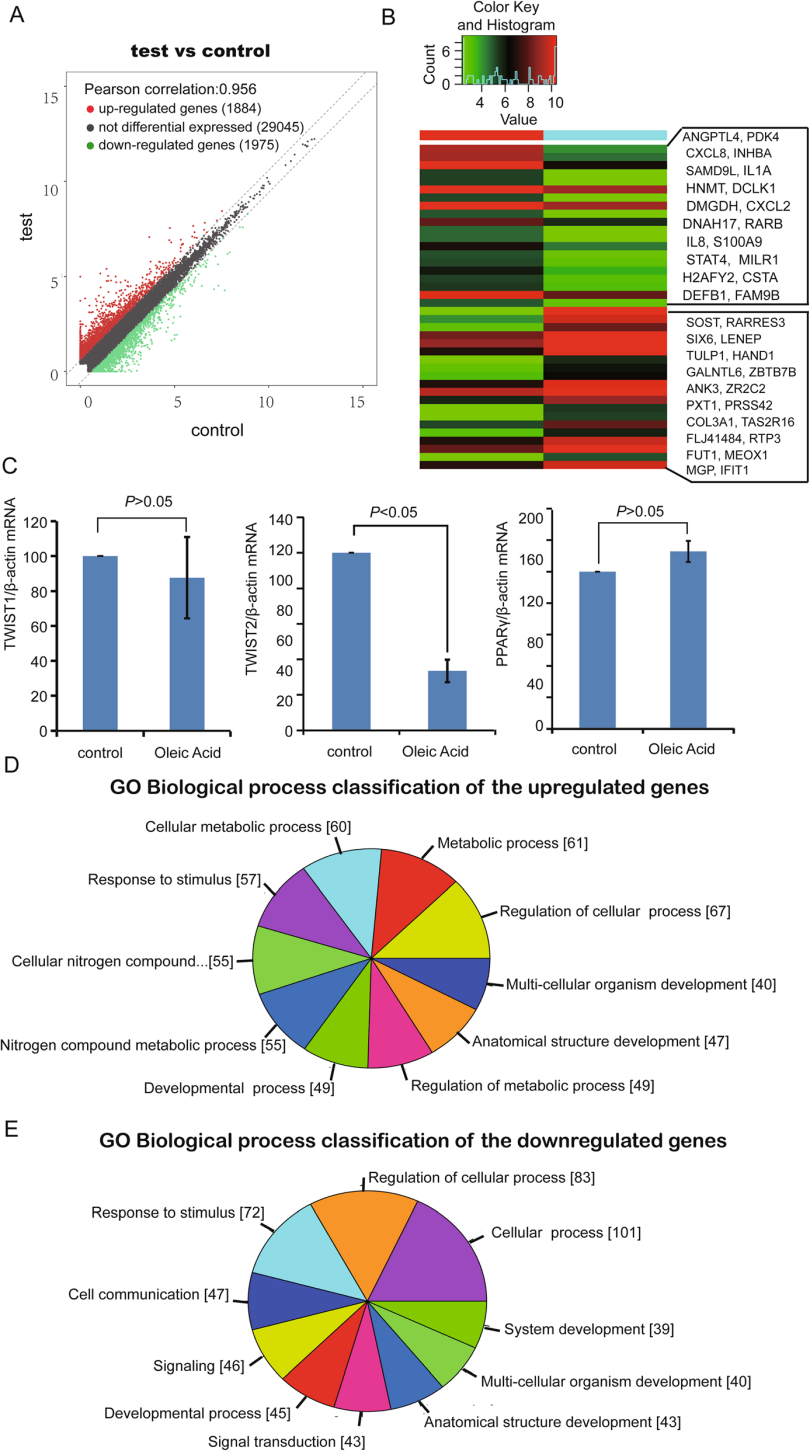
RNA sequencing analysis of transcriptional changes and GO biological process analysis in cultured LO-2 cells treated with OA (50 μg/mL, 48 h). **a** After sequencing analysis, the mRNA transcriptional changes between the control and test groups were analyzed. Shown here is a scatter map of mRNA transcriptional changes. **b** The top 20 upregulated and downregulated genes in the OA treatment group were selected and are shown in a heat map. **c** RT-PCR was conducted to determine TWIST1, TWIST2 and PPARγ mRNA changes in response to OA treatment as described in the methods section. The results are shown in the bar graphs. **d** GO biological process analysis was conducted, and the classifications of the upregulated genes are shown. **e** The classifications of the downregulated genes for GO biological processes are shown

RNA sequencing of LO-2 cells treated with OA for 48 h showed that 1884 genes were upregulated and 1975 genes were downregulated in the OA treatment group compared with the control group (Fig. 3a). Cluster analysis of the top 20 upregulated and downregulated genes is shown in Fig. 3b. TWIST1 and PPARγ mRNA levels showed no significant difference (*P* > 0.05) between the groups in the RNA sequencing results, but TWIST2 was downregulated significantly with OA treatment (*P* < 0.05) (Fig. 3c). Further GO enrichment analysis of the RNA-seq data revealed 542 differentially expressed (DE) upregulated genes and their associated biological processes (BPs) (Fig. 3d) and 514 DE downregulated genes and associated BPs (Fig. 3e). The details of the top 20 upregulated and downregulated genes under oleic acid treatment by RNA sequencing are shown in Table 3.

**Table 3:** Details on the top 20 upregulated and downregulated genes under oleic acid treatment as determined by RNA sequencing. The top 20 upregulated genes are listed, including, among others, ASNS-209, APLP2–202, and PSAP-201. The top 20 downregulated genes included, among others, APLP2–201, DDX27–206, and CARD10–203. All the genes were significantly differentially expressed (*P* < 0.05)

Gene name	Transcript name	log_**2**_(fold change)	Fold change	***P*** value	Gene name	Transcript name	log_**2**_(fold change)	Fold change	***P*** value
**Upregulated genes**					**Downregulated genes**				
ASNS	ASNS-209	4.9946	31.8794	0.0000	APLP2	APLP2–201	−3.2019	0.1087	0.0001
APLP2	APLP2–202	4.0958	17.0984	0.0070	DDX27	DDX27–206	− 3.1990	0.1089	0.0193
PSAP	PSAP-201	4.0106	16.1183	0.0283	CARD10	CARD10–203	−2.9708	0.1276	0.0235
EIF4G1	EIF4G1–221	3.7234	13.2089	0.0349	JADE3	JADE3–203	− 2.7405	0.1496	0.0000
ANGPTL4	ANGPTL4–202	3.5879	12.0241	0.0008	APOL2	APOL2–201	−2.7045	0.1534	0.0246
NDUFS8	NDUFS8–210	3.3598	10.2658	0.0235	POLR2A	POLR2A-201	−2.5492	0.1709	0.0266
POLR2B	POLR2B-213	3.3478	10.1812	0.0213	KIF14	KIF14–202	−2.5153	0.1749	0.0272
SERPINE2	SERPINE2–203	3.3009	9.8552	0.0139	CD59	CD59–203	−2.4796	0.1793	0.0002
HAX1	HAX1–202	3.1570	8.9197	0.0238	KATNA1	KATNA1–205	−2.3728	0.1931	0.0247
CCDC47	CCDC47–207	3.1261	8.7309	0.0229	EIF4G1	EIF4G1–216	−2.3661	0.1940	0.0057
TIPARP	TIPARP-206	3.0047	8.0262	0.0214	POLR2B	POLR2B-205	−2.3462	0.1967	0.0220
ANGPTL4	ANGPTL4–201	2.9858	7.9219	0.0001	ATP5G2	ATP5G2–204	−2.3328	0.1985	0.0145
UBA2	UBA2–208	2.8114	7.0199	0.0272	NOP2	NOP2–203	−2.3103	0.2016	0.0275
EIF4G1	EIF4G1–207	2.6434	6.2481	0.0280	GPANK1	GPANK1–201	−2.2093	0.2162	0.0022
PPP2R2A	PPP2R2A-201	2.5557	5.8797	0.0221	SEC22C	SEC22C-205	−2.1893	0.2193	0.0258
PDK4	PDK4–201	2.5308	5.7790	0.0000	ALPI	ALPI-201	−2.1868	0.2196	0.0045
ZFAND2B	ZFAND2B-206	2.4906	5.6202	0.0000	CTSA	CTSA-201	−2.1791	0.2208	0.0134
DDX19B	DDX19B-208	2.4219	5.3586	0.0370	ALDH3B1	ALDH3B1–208	−2.1746	0.2215	0.0139
ZYX	ZYX-203	2.4101	5.3151	0.0480	MEOX1	MEOX1–202	−2.1356	0.2276	0.0000
WASHC5	WASHC5–202	2.2769	4.8462	0.0226	SNX3	SNX3–204	−2.1237	0.2295	0.0156

### Genes in the PPAR signaling pathway were enriched significantly based on RNA sequencing

**Fig. 4:**
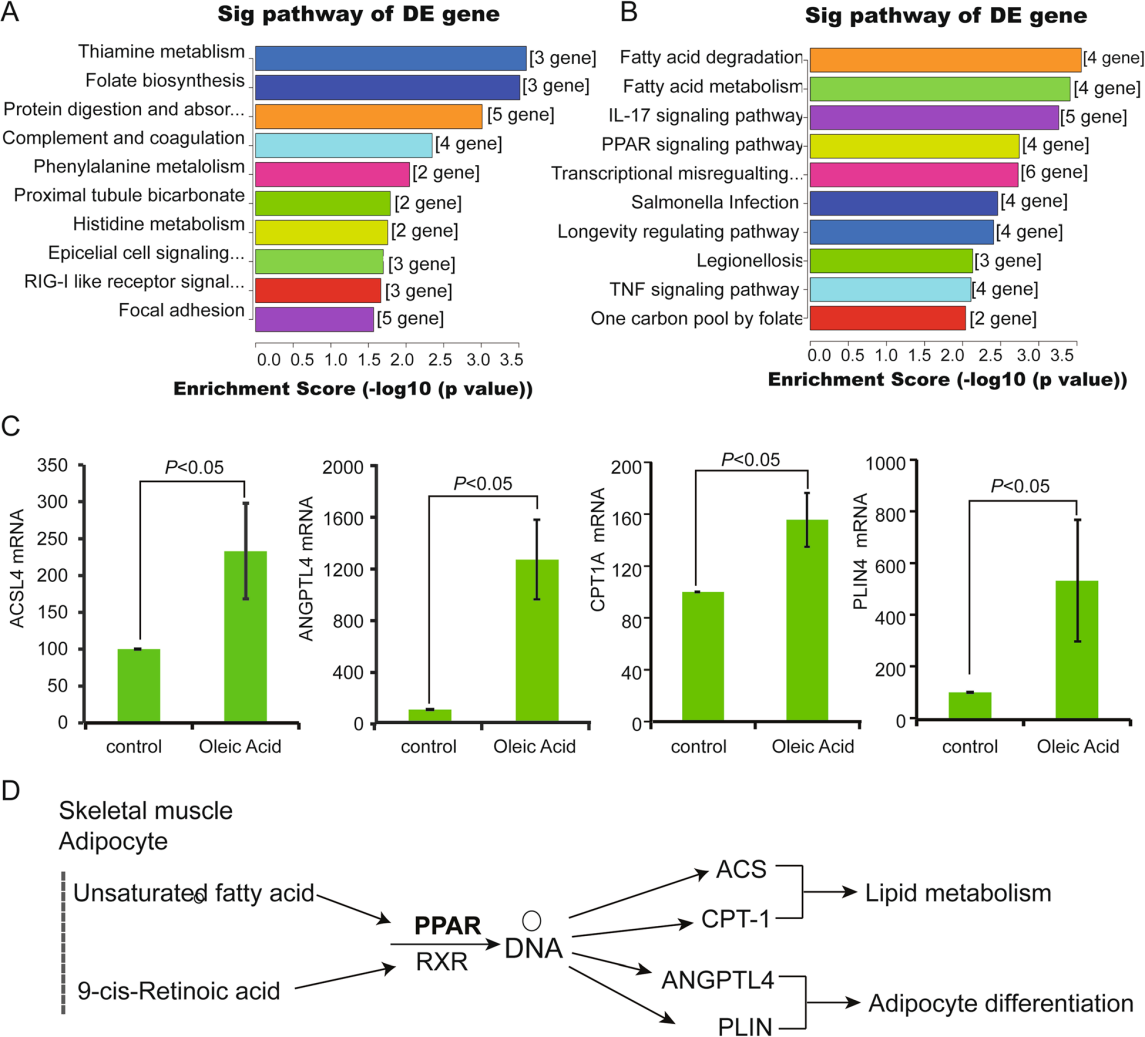
PPAR signaling pathway genes were enriched significantly in LO-2 cells by OA treatment (50 μg/mL, 48 h). **a** KEGG pathway analysis based on the KEGG website. The top 10 upregulated pathways of differentially expressed (DE) genes are shown here. **b** The top 10 downregulated pathways of DE genes based on the KEGG website shown here. **c** The mRNA levels of four genes in the PPAR signaling pathway, namely, ACSL4, ANGPTL4, CPT1A, and PLIN4, showed increased transcription based on RT-PCR. **d** A schematic model of the PPARγ pathway in lipid metabolism and adipocyte differentiation was drawn, indicating the mechanism of the PPAR signaling pathway in NAFLD

Compared with the control, the DE genes in OA-treated LO-2 cells (50 μg/mL, 48 h) were involved in 62 pathways, with 42 upregulated (top 10 shown in Fig. 4a) and 20 downregulated (top 10 shown in Fig. 4b). In the top 10 upregulated pathways of DE genes, the IL-17 signaling pathway [hsa04657], TNF signaling pathway [hsa04668], and PPAR signaling pathway [hsa03320] were listed. Four genes in the PPAR signaling pathway were upregulated in OA (50 μg/mL, 48 h)-treated cells (*P* = 0.0018). These four genes are acyl-CoA synthetase long chain family member 4 (ACSL4), angiopoietin like 4 (ANGPTL4), carnitine palmitoyltransferase 1A (CPT1A), and perilipin 4 (PLIN4). RT-PCR verified their up regulation (Fig. 4c). A schematic model for the PPAR function is proposed, as shown in Fig. 4d. During adipogenesis, PPAR can be induced by unsaturated fatty acids or 9-cis-retinoic acid and can upregulate ACSL4, ANGPTL4, CPT1A, and PLIN, which can regulate lipid metabolism or adipocyte differentiation.

## Discussion

The present study focuses on the role of TWIST1, TWIST2 and PPARγ in nonalcoholic steatohepatitis progression. To do this, the serum protein levels of TWIST1, TWIST2, and PPARγ in the serum of NAFLD patients were examined with varying stages of disease, and significant changes in the TWIST proteins were noted. In vitro and in vivo models of the disease were also studied, and TWIST2 seemed to be affected the most in these models. The results using each of these models provide additional evidence that the TWIST2 transcription factor may play a role in NAFLD.

TWIST2 seems to be particularly important in NAFLD because the results and the publication of a recent article showed that TWIST2 was involved in the development of NAFLD [16]. TWIST2 is a basic helix-loop-helix (bHLH) transcription factor that is highly related to TWIST1 and appears to have some redundant and nonredundant functions [17]. TWIST transcription factors bind as homodimers or heterodimers to E-box consensus sites and are transcriptional activators or repressors depending on the cellular context [17, 18]. TWIST proteins have been extensively studied for their oncogenic properties and their role in the epithelial-to-mesenchymal transition (EMT), but their involvement in other processes is less well known [19]. *twist2*-null mice have many abnormalities in fat and glucose metabolism and are presumed to die soon after birth because of the upregulation of cytokines in an NF-κB-dependent manner [20]. A decrease in TWIST2 protein in the in vitro and in vivo models of NAFLD was found. Interestingly, a decrease in TWIST2 protein levels in the serum of NAFLD patients at all stages of the disease was also noticed. This suggests that in NAFLD, a reduction in the level of TWIST2 protein may be an early event that increases inflammation. It also suggests that TWIST2 protein levels in serum may be a biomarker of NAFLD. It has recently been shown that TWIST1 mRNA and protein can be found in exosomes secreted from hepatic stellate cells (HSCs) and that the levels of TWIST1 mRNA and protein decrease in models of hepatic injury and fibrosis [21]. It is likely that TWIST2 is also secreted from the liver compartment and that changes in TWIST2 protein content can be indicative of a developing disease process.

TWIST1 serum protein levels varied greatly with the severity of disease, with significantly lower levels in the mild fatty liver group and significantly higher levels in the moderate fatty liver group. The level of TWIST1 protein in the severe fatty liver group was the same as that in the control group. This pattern of TWIST1 serum protein may indicate complex regulation of the TWIST1 gene during the development of NAFLD. TWIST1 was considered an anti-inflammatory marker, as low mRNA and protein expression of TWIST1 was associated with increased expression of proinflammatory cytokines and decreased insulin sensitivity in humans [22]. Lower levels of TWIST1 in the early stages of NAFLD may lead to increased inflammation and disease progression.

Increased protein expression of PPAR is a general property of steatotic livers. The contribution of PPAR is to the maintenance of a steatotic phenotype in liver cells [23]. PPARγ is capable of activating the expression of genes involved in TG accumulation in hepatocytes and promoting the generation of fatty liver [24]. While no change was seen in PPAR*γ* protein levels in the patient serum or in the in vivo and in vitro models, the RNA sequence analysis of OA-treated cells suggested that the PPAR signaling pathway was involved in the hepatic steatosis process. It is believed that PPAR may regulate downstream lipid metabolism or adipocyte differentiation through ACSL4, ANGPTL4, CPT1A, and PLIN4. Further investigation into the mechanism of how PPAR contributes to NAFLD is warranted.

Chronic inflammation has also been implicated in the development and progression of NAFLD [25, 26]. TWIST1 and TWIST2 have been shown to reduce inflammation by inhibiting the NF-kB pathway [18, 20]. As mentioned, twist-2-deficient mice produce high levels of cytokines [20]. An interesting finding of the GO analysis of the RNA sequencing data revealed an enhanced IL-17 signaling pathway in OA-treated cells. IL-17 signaling has become of greater significance in NAFLD in recent years [27]. An enhancement in the TNF signaling pathway was also found, another important inflammatory pathway implicated in NAFLD [28–30]. It has been reported that a significantly higher concentration of TNF-alpha is associated with both the type of inflammatory cells and with increased vascularization in various tissue types [31].

### Study strength and limitations

The present findings prompt TWIST2 as a potential new biomarker for the early detection of NAFLD. Meanwhile, the involvement of the PPAR signaling pathway hints further perspective in hepatocytes steatosis. However, there are some limitations in this study. Although the function of PPAR is involved in steatosis, the mechanism of how PPAR contributes to NAFLD is unclear. It is necessary to determine the PPAR gene function and to study the phenotype if we block the PPAR signaling pathway. Also, the inconsistence of TWIST1 gene expression at protein level between in vitro, in vivo and in patient samples is needed to be fully examined. The mechanism of downregulation of the TWIST2 gene expression in 3rd month is also needed to be understood. Together this paper demonstrates the importance of the TWIST2 gene in the clinical prognosis and roles of PPAR and TWIST1 in NAFLD development needed fully investigated.

## Conclusions

In summary, the present study shows that the TWIST2 and PPAR signaling pathways are important in NAFLD and sheds light on a potential mechanism of steatosis. The present findings establish TWIST2 as a new biomarker for the early detection of NAFLD. A simple blood test for TWIST2, a potential biomarker, may allow one to detect the early stages of NAFLD. Future studies on the PPAR signaling pathway will be urgent to clarify the roles of these genes in NAFLD.

## Data Availability

The data will be available on request.

## Abbreviations

TWIST1: Twist family bHLH transcription factor 1
TWIST2: Twist family bHLH transcription factor 2
PPARγ: Peroxisome proliferator activated receptor gamma
NAFLD: Nonalcoholic fatty liver disease
ELISA: Enzyme-linked immunosorbent assay
IL-17: Interleukin (IL)-17
TNF: Tumor necrosis factor
IR: Insulin resistance
NASH: Nonalcoholic steatohepatitis
VLDL: very Low density lipoprotein
EMT: Epithelial-to-mesenchymal transition
IPGTT: Intraperitoneal glucose tolerance test
IPITT: Intraperitoneal insulin tolerance test
H&E: Hematoxylin and eosin
PBS: Phosphate-buffered saline;
RIPA: Radioimmunoprecipitation assay
GO: Gene Ontology
KEGG: Kyoto Encyclopedia of Genes and Genomes
BMI: Body mass index
HOMA: Homeostasis model assessment
DE: Differentially expressed
ACSL4: Acyl-CoA synthetase long chain family member 4
ANGPTL4: Angiopoietin like 4
CPT1A: Carnitine palmitoyltransferase 1A
PLIN4: Perilipin 4
HSCs: Hepatic stellate cells
